# Immune profiling links autoimmune hepatitis to human herpesvirus 6 and relaxin receptor antigens

**DOI:** 10.1084/jem.20250959

**Published:** 2026-03-03

**Authors:** Arielle Klepper, James Asaki, Colette M. Caspar, Andrew F. Kung, Sara E. Vazquez, Aaron Bodansky, Anthea Mitchell, Sabrina A. Mann, Kelsey Zorn, Isaac Avila-Vargas, Swathi Kari, Melawit Tekeste, Javier Castro, Briton Lee, Maria Duarte, Mandana Khalili, Monica Yang, Paul Wolters, Jennifer Price, Emily Perito, Sandy Feng, Jacquelyn J. Maher, Michael R. Wilson, Jennifer C. Lai, Christina Weiler-Normann, Ansgar W. Lohse, Joseph DeRisi, Michele May-Sien Tana

**Affiliations:** 1 Department of Medicine, University of California, San Francisco, San Francisco, CA, USA; 2 Department of Biochemistry, University of California, San Francisco, San Francisco, CA, USA; 3 Department of Dermatology, Mass General Hospital, Boston, MA, USA; 4 Department of Pediatrics, University of California, San Francisco, San Francisco, CA, USA; 5 Chan Zuckerberg Biohub, San Francisco, CA, USA; 6 Department of Surgery, University of California, San Francisco, San Francisco, CA, USA; 7 Department of Medicine, University Medical Center Hamburg-Eppendorf, Hamburg, Germany; 8 UCSF Liver Center, University of California, San Francisco, San Francisco, CA, USA; 9 Gladstone-UCSF Institute of Genomic Immunology, San Francisco, CA, USA

## Abstract

Autoimmune hepatitis (AIH) is a severe, chronic disease where IgG elevation and autoantibody profile are defining features. However, linking autoantibodies to AIH pathogenesis remains elusive. We employed phage-display immunoprecipitation sequencing and uncovered a novel humoral signature specific to AIH. Embedded within this signature were antibodies against the known AIH autoantigen SLA/LP and novel reactivities to disco-interacting protein 2 homolog A (DIP2A), and the relaxin family peptide receptor 1 (RXFP1). Fine mapping of the DIP2A epitope revealed preferential enrichment for a nearly identical 9–amino acid sequence derived from the U27 protein of human herpesvirus 6 (HHV6). Preincubation with the HHV6 epitope blocked DIP2A binding, consistent with cross-reactivity. AIH patients positive for anti-DIP2A had higher titers of HHV6 IgG, suggestive of reactivation. AIH patients had antibodies against the antifibrotic receptor, RXFP1, which inhibited relaxin-2 signaling in an IgG-dependent manner. These data provide evidence for a novel serological profile in AIH, linking HHV6 reactivation anti-RXFP1 antibodies to disease pathogenesis.

## Teaser

Patients with autoimmune hepatitis have a unique autoantibody signature, yielding clues about disease pathophysiology, including evidence for cross-reactivity with a common herpesvirus, human herpesvirus 6, and antibodies targeting the antifibrotic relaxin receptor with potential to promote fibrosis progression.

## Introduction

Autoimmune hepatitis (AIH) is a chronic, severe liver disease identified in the 1950s, affecting all ages, rising in incidence (Lamba et al., 2021), and disproportionately impacting people of color (Lee et al., 2018; Wen et al., 2018). Treatment of AIH frequently requires lifelong therapy with immunosuppressive medications, with multiple morbid side effects. Despite the long-standing clinical burden of AIH, little is known about the etiopathogenesis of disease. Clinically, AIH onset can be marked by an episode of acute hepatitis. Initial workup includes assessment of total IgG levels (commonly elevated in patients with AIH), as well as evaluation for characteristic autoantibodies, particularly antinuclear antibodies (ANA), anti-smooth muscle antibodies, and liver-kidney microsomes type 1 (anti-LKM-1), none of which are specific to AIH or to the liver itself, as well as anti-soluble liver antigen and liver pancreas (SLA/LP), a liver antigen highly specific to AIH, present in up to 20% of AIH patients (Kanzler et al., 1999; Manns et al., 1987; Wies et al., 2000). Determination of this serologic profile can help to discriminate between subtypes of AIH types, AIH-1 (primarily affecting adults) and AIH-2 (primarily affecting children). However, the significance of autoantibodies in determining prognosis, or their role in disease pathogenesis, is debated.

The pathogenesis of AIH is believed to result from a combination of genetic, immunologic, and environmental factors. Regarding genetic predisposition, genome-wide association studies of patients from Europe and North America have shown a significant association between AIH-1 and HLA alleles DRB1*0301 and DRB1*0401 (de Boer et al., 2014). Several studies have further implicated a breakdown in self-tolerance as a core immunologic mechanism of disease (Longhi et al., 2004; Ferri et al., 2010). Numerous environmental triggers have also been associated with AIH, such as medications and viruses, for example, minocycline, nitrofurantoin, hepatitis viruses, and human herpesviruses (Zachou et al., 2021; Manns et al., 2015). However, a driving, central autoantigen in AIH-1 has not been identified, and in contrast, the disease is actually characterized by a polyreactive immunoglobulin profile (Taubert et al., 2022). Several groups have linked infections to the development of AIH. However, molecular mimicry as a pathogenic mechanism of disease remains controversial.

While autoantibodies play a central role in AIH diagnosis clinically, in-depth analysis of the pathogenic role of B cells and antibodies has not been extensively pursued (Kramer et al., 2025), and has been cited as a core goal of the AIH research agenda by professional societies (Sebode et al., 2018). Phage-display immunoprecipitation sequencing (PhIP-seq) is a platform developed to perform an unbiased, high-throughput assessment of antibodies across a broad array of autoimmune conditions (Zamecnik et al., 2024; Larman et al., 2011; Vazquez et al., 2020; Vazquez et al., 2022; O’Donovan et al., 2020; Rackaityte et al., 2023; Bodansky et al., 2024a; Mandel-Brehm et al., 2019; Mandel-Brehm et al., 2022). We applied PhIP-seq to study serum or plasma from 115 AIH patients obtained from multicenter, international collaborative cohort of patients, and compared these results with a robust series of 303 control serum or plasma samples from patients with metabolic-associated steatotic liver disease (MASLD), primary biliary cholangitis (PBC), rheumatoid arthritis (RA), or healthy controls to further define novel disease- and tissue-specific autoantibody targets to better inform our understanding of AIH pathogenesis.

## Results and discussion

### Autoantibody testing was performed on well-characterized, multicenter, international cohorts with robust controls

As part of a multicenter, international collaboration, specimens of serum or plasma from patients with AIH (*n* = 115), MASLD (*n* = 178), PBC (*n* = 26), RA (*n* = 5), and systemic sclerosis (SSc)-interstitial lung disease (SSc-ILD, *n* = 30) were contributed by four well-characterized patient cohorts: Prospective Observational Study to Understand Liver Diseases (POSULD, San Francisco General Hospital, San Francisco, CA, USA), FrAILT (University of California, San Francisco [UCSF] Parnassus Hospital, San Francisco, CA, USA), the Eppendorf University cohort (Hamburg, Germany), and the UCSF ILD cohort (UCSF Parnassus Hospital, San Francisco, CA, USA). Deidentified healthy control samples were obtained from two sources: the New York Blood Center or Food and Drug Administration (FDA)-licensed blood collection facilities purchased through SeraCare (K2EDTA human plasma). Clinical characteristics of the patients run on PhIP-seq are summarized in Tables 1 and 2.

**Table 1.** Characteristics of the study population assayed by PhIP-seq

Diagnosis	*n*	Mean age	Female (%)	Caucasian (%)	Latinx (%)	Advanced fibrosis (%)
AIH	115	58	70	96	14	60
MASLD	178	60	60	82	67	63
PBC	26	58	94	84	21	58
RA	5	–	–	–	–	–
Healthy	94	–	–	–	–	–

**Table 2.** Characteristics of AIH patients assayed by PhIP-seq (*n* = 115)

​	Mean ALT	Mean AST	Mean IgG	Advanced fibrosis (F3/F4)	Steroid-containing regimen	Nonsteroid regimen	No reported treatment^a^	Anti-SMA positive
AIH	79	66	1,520	58%	46%	34%	20%	81%

Anti-SMA, anti-smooth muscle antibodies.

a 6% of patients were reported as being off all treatment, and an additional 14% of patients did not have a treatment regimen reported.

### A signature for AIH disease status includes both novel and previously recognized antibody targets in AIH

**Figure 1.**
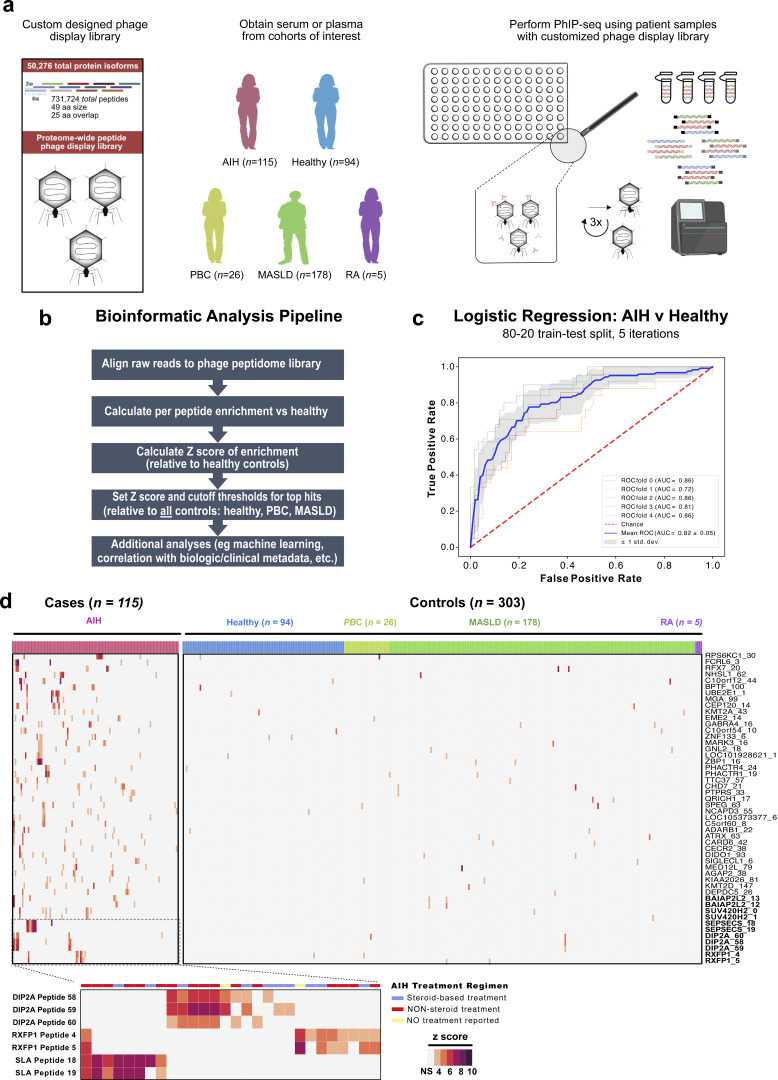
**PhIP-seq** **enables AIH disease prediction and identifies both known and novel highly specific autoantibody targets. (a)** Phage-display library design, and an overview of methods to apply PhIP-seq to evaluate the AIH and control cohort. **(b)** Summary of the customized bioinformatics analysis pipeline applied to analyze next-generation sequencing output of PhIP-seq data. **(c)** ROC curve analysis for prediction of AIH versus all control patients, noting AUC. **(d)** Heatmap of significantly enriched peptides, which are highly specific to AIH (>99% specific). Depicted in the heap map are top hits where multiple overlapping peptides were immunoprecipitated by PhIP-seq (peptide name at bottom, right axis). The top legend indicates whether samples correspond to case patients (AIH: pink) or controls (healthy controls: blue; PBC: yellow; MASLD: green; RA: purple); boxes are shaded by Z-score of enrichment (legend bottom left), and Z <3 is shaded gray. Highlighted on the right axis, in bold, are the hits with the highest mean Z-scores across all enriched peptides. The panel below zooms in on results of Z-score of enrichment for the AIH patients positive for any of the top targets in bold, with a top legend indicating their AIH treatment regimen (red: on treatment without steroids; blue: on treatment containing steroids; yellow: no reported treatment). ROC curve, receiver operating characteristic curve.

A schematic overview of PhIP-seq methodology is summarized in Fig. 1 a, as well a workflow for the customized bioinformatics approach to analysis of these data (Fig. 1 b). Applying this workflow, we performed logistic regression on peptide enrichment, calculated by comparing patient enrichment relative to the mean of healthy control signal for that peptide (further detailed in Materials and methods). Logistic regression was performed with fivefold cross-validation with an 80/20 train/test split (Fig. 1 c). The resulting model was able to classify a diagnosis of AIH (versus all non-AIH controls) on the basis of PhIP-seq autoreactivity against all peptides (731,724 peptides; mean area under the curve [AUC] = 0.82). These results were significant, given that serologic diagnosis of AIH is not only heterogeneous—and may include patients who present without positive autoantibodies at the time of clinical assessment (Lohse and Hennes, 2007)—but also that the signature retains predictive power even when applied to patients on immunosuppressive therapy for AIH (80% of AIH patients studied; summarized in Table 2). This aligns with findings from a group that treating patients with steroids or rituximab has only a minimal effect on the autoreactome detectable by PhIP-seq (Bodansky et al., 2024b). When limiting the logistic regression model to a smaller number of features using only the top 100 most enriched peptides (when ranked by sum of reads per 100,000 [RPK]), predictive power was degraded (AUC = 0.62), confirming the presence of substantial heterogeneity in the autoreactivity profiles of individual AIH patients. This is consistent with prior reports that a polyreactive immunoglobulin response is a defining feature of AIH (Taubert et al., 2022).

Because overfitting is a concern for machine learning techniques, it is important to identify informative features of the model and orthogonally validate them biochemically. We focused our analysis on AIH-specific and significantly enriched targets, which were selected by setting a Z-score threshold of >3 relative to the mean of control samples, and further requiring that hits could not be significantly enriched in >1% of all controls (MASLD, PBC, RA, and healthy controls, ≤3/303 patients, defined as 99% specific) and must be enriched in at least 5% of the AIH patients (>6/115 patients). This stringent approach identified 50 hits at the peptide level, representing 44 genes. The top 50 peptides, their corresponding amino acid sequence, and the RefSeq and/or UniProt ID can be found in Table S1. None of the top 50 hits were observed to be significantly enriched in prior studies from our group on autoimmune polyendocrine syndrome type 1, in which AIH is a known manifestation (Vazquez et al., 2020).

Among the top 50 hits in AIH, five proteins were represented by multiple peptides (peptides in bold, Fig. 1 d); given this internal validation, we focused on the top three most enriched targets (by Z-score) from this group for further study: SEPSECS (also referred to as SLA), which is clinically utilized and well-characterized AIH autoantigen, the relaxin family peptide receptor 1 (RXFP1), and the disco-interacting protein 2 homolog A (DIP2A), displayed in the bottom panel (Fig. 1 d).

To evaluate the possibility that PhIP-seq–detected autoreactivities are a surrogate for other clinical features, we compared antibody enrichment against our top three hits with clinical data. Metadata variables included IgG titers, immune suppression, and disease activity. The analysis of variance (ANOVA) for AIH patients is shown in Table 2 (for complete ANOVA results, see Table S2) and included a composite measure of “biochemical remission” defined as patients with normal AST, ALT, and IgG levels. Notably, IgG induction was not associated with enrichment in antibodies against any of the top three peptides (Table S2). However, patients who did not achieve biochemical remission had significantly greater SEPSECS/SLA antibody enrichment (false discovery rate [FDR]–adjusted P < 0.01, Table 3). This finding is consistent with previously reported data that SLA-positive AIH patients require longer durations of therapy to achieve a first complete response (Zachou et al., 2020), while other SEPSECS/SLA trends did not maintain significance when correcting for multiple hypothesis testing (Table 3). Positivity for DIP2A and RXFP1 was not significantly associated with any of the available clinical metadata variables, other than having AIH (Table S2).

**Table 3.** Significant correlations between AIH patient metadata (Table 2) and top PhIP-seq peptides (peptides included in analysis are summarized in Fig. 1 d, bottom panel: SEPSECS/SLA peptides 18 and 19; DIP2A peptides 58, 59, and 60; and RXFP1 peptides 4 and 5)

Peptide (outcome)	Metadata (predictor)	F value	P value	P value, adjusted^a^
SEPSECS/SLA peptide 19	AIH not in biochemical remission	14.24	0.00027865	0.00906634
SEPSECS/SLA peptide 18	AIH not in biochemical remission	13.92	0.0003238	0.00906634
SEPSECS/SLA peptide 19	Nonsteroid treatment regimen	8.15	0.00527844	0.07389812
SEPSECS/SLA peptide 18	Nonsteroid treatment regimen	8.40	0.00465401	0.07389812

a Benjamini–Hochberg correction was applied to control for multiple hypothesis testing.

### PhIP-seq identifies all patients clinically positive for anti-SLA antibody, and provides finer mapping of the known SLA epitope

**Figure 2.**
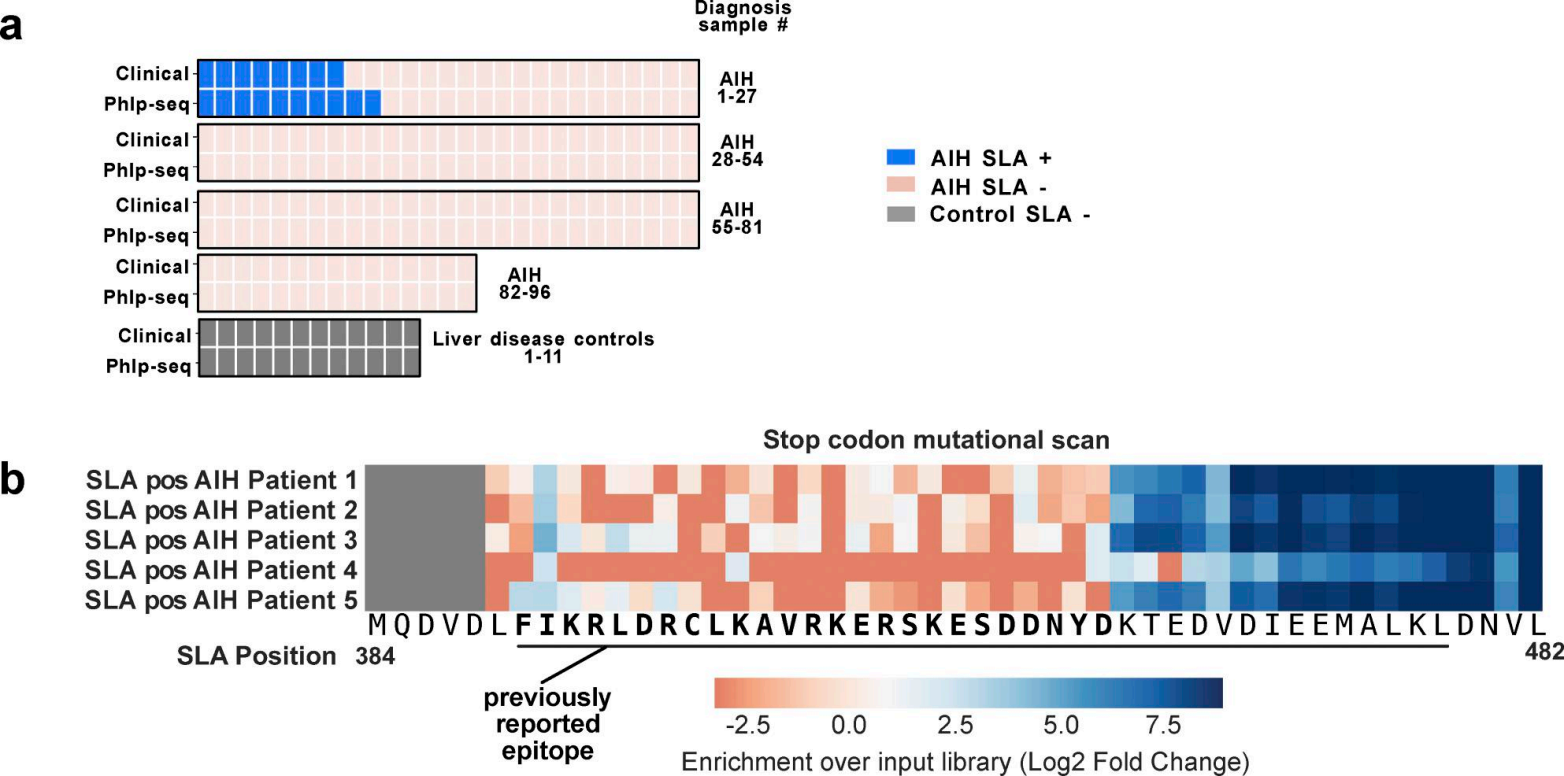
**PhIP-seq is highly concordant with clinical results for the anti-SLA antibody, and yields finer mapping of the known SLA epitope by PhASER. (a)** Heatmap comparing all patients for which clinical anti-SLA testing results were available (96 AIH patients, 11 controls). Patients positive for anti-SLA antibody by PhIP-seq (blue, bottom row) were all positive by clinical testing (blue, top row), and two additional positive patients were detected by PhIP-seq. SLA-negative AIH patients are noted in beige, and non-AIH patients were noted in gray, all of whom were negative for anti-SLA antibody using both assays. **(b)** Heatmap of SLA/LP-positive (pos) AIH patient reactivity (*n* = 5) to SLA/LP peptide 18 (peptide sequence at the bottom of the heatmap) with stop-codon substitutions at each position, starting at SLA/LP amino acid 389. The sequence of the previously reported minimal SLA/LP epitope is underlined. Reactivity to SLA/LP became detectable (blue) after the peptide extended through position 414, and was not detected with shorter peptide truncations (orange).

Given that one of the top three autoantibodies identified by PhIP-seq targets the known autoantigen SEPSECS/SLA, we compared our results with clinical data for SLA-positive autoantibodies. A single PhIP-seq peptide (SEPSECS_18, Table S1) can identify 100% of patients that tested positive via the clinical assay. In addition to detecting SLA positivity in every patient who clinically tested positive for the antibody, we also detect SLA autoreactivity in two additional AIH patients who were not detected using the clinical assay (Fig. 2 a). Notably, no patients without AIH were positive for the SLA-reactive peptide by PhIP-seq among our 303 controls (Fig. 1 d), indicating remarkable specificity for a single peptide biomarker. Of the enriched peptides, both SEPSECS fragments correspond to the region previously reported to be essential for antibody reactivity to the AIH autoantigen SLA/LP (Wies et al., 2000). The peptides used as bait in the PhIP-seq assay are 49 amino acids in length. To more precisely map the antibody epitopes, a second Phage-Assisted Scanning Epitope Recovery (PhASER) PhIP-seq library was created to probe the determinants of antibody binding within selected peptides. To identify the downstream boundary of the SLA/LP epitope, the library included sequential stop-codon substitutions spanning the critical region of reactivity (PhIP-seq SLA/LP peptide 18), employing a similar approach used in our previous investigation of the anti-Hu epitope (O’Donovan et al., 2020). Antibody reactivity to the SLA/LP peptide sequence became detectable when the peptide extended through amino acid 414, defining the right-most boundary of the epitope (Fig. 2 b). This result confirmed the previously reported minimal antigen, and further refined it to a 25–amino acid stretch (389–414).

### Antibodies against DIP2A are highly specific to AIH, and the target epitope derives from human herpesvirus 6

**Figure 3.**
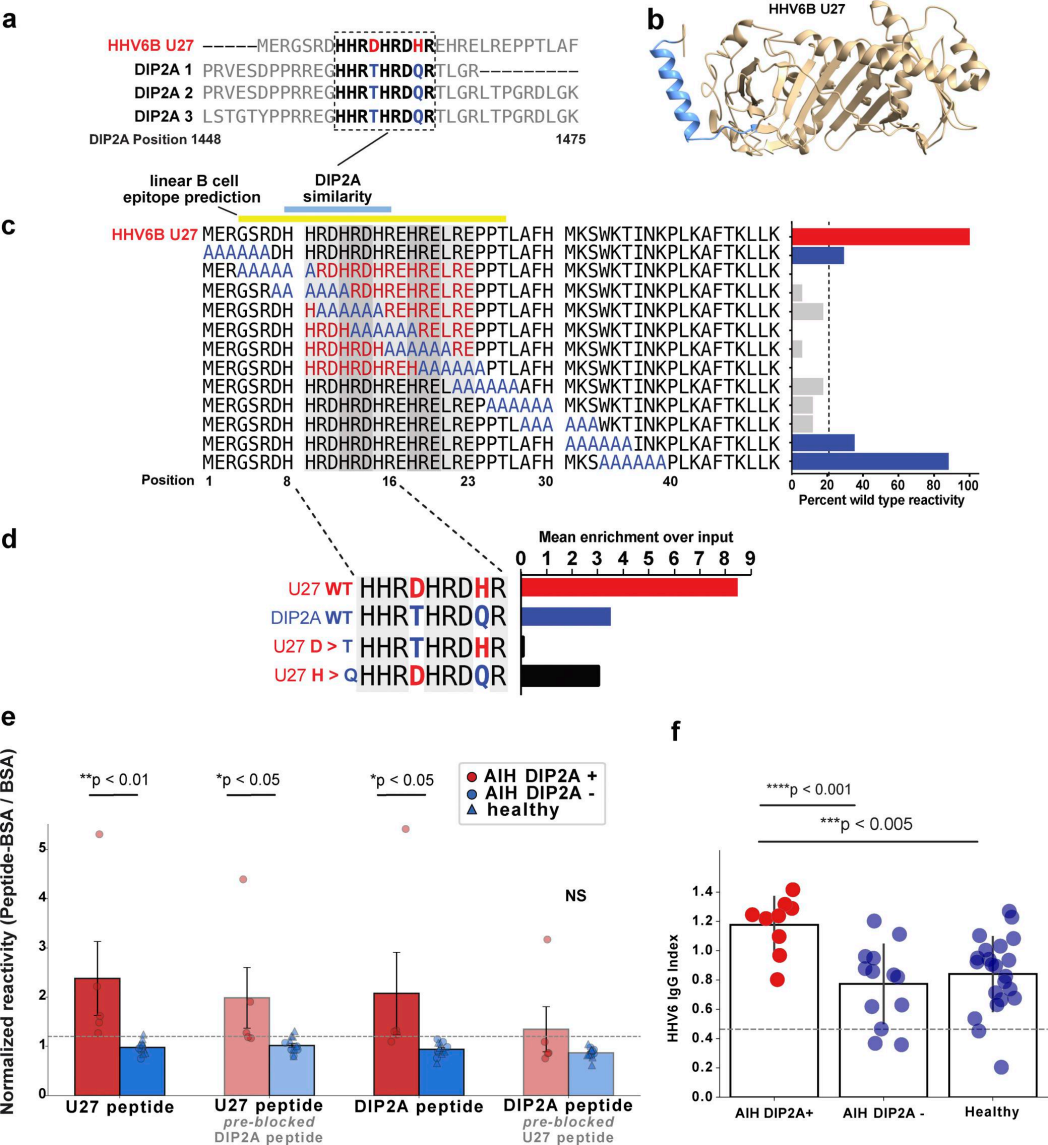
**BLAST uncovers reactivity to a distinct region of the HHV6 U27 protein with sequence similarity to DIP2A. (a)** BLAST search results demonstrating alignment of DIP2A peptide hits to HHV6 U27 protein, 7/9 amino acids were identical, and among nonidentical amino acids from U27 (red), only T (blue in DIP2A) → D (red in U27) is a nonconservative substitution. **(b)** HelixFold rendering of HHV6 U27 protein, with the BepiPred-predicted linear epitope highlighted in blue. **(c)** HHV6 alanine scanning schematic (left) and resulting impact on HHV6 reactivity (right); reactivity is normalized to percentage of wild-type reactivity (red bar). Alanine mutants with <20% of wild-type reactivity (gray bars) indicate U27 amino acids 4–30 as the critical region for antibody reactivity, whereas mutations outside this region (blue bars) had a lesser impact on reactivity. Locations of the BepiPred-predicted linear epitope (yellow), DIP2A similarity (blue), and immunoproteasome processing (orange) all converge on this region of reactivity. **(d)** Point mutations of the nonidentical residues in HHV6 (red) and DIP2A (blue); when reacted with AIH patient serum (*n* = 2), the wild-type HHV6 sequence leads to 8× enrichment over input library, more than twice that of DIP2A wild-type sequence. Mutating the D found in the HHV6 wild-type sequence (red) to the T found in DIP2A (blue; a nonconservative change) abolished this effect. **(e)** Luminex-based orthogonal validation assay confirms significant reactivity to the HHV6 U27 and DIP2A peptides in AIH patients positive for the DIP2A antibody by PhIP-seq (red bar/circles) compared with AIH patients negative for DIP2A by PhIP-seq (blue circle) or healthy controls (blue triangle); gray dashed line indicates mean + 2 SD of DIP2A-negative patients and healthy controls. Normalized reactivity (peptide–BSA/BSA alone) is plotted on the y axis. Preblocking with the putative cross-reactive peptide (lightly shaded bars) decreased reactivity and, in the case of HHV6 preincubation, led to loss of significant DIP2A reactivity (NS, far right). **(f)** HHV6 IgG index in AIH patients positive for DIP2A (red circles) compared with AIH patients negative for DIP2A (blue circles, middle), and healthy controls (blue circles, right); the assay cutoff for positivity is denoted by the dashed gray line; AIH patients positive for DIP2A by PhIP-seq had significantly higher HHV6 IgG titers than AIH patients negative for DIP2A (P < 0.001) or healthy controls (P < 0.005).

While the hepatic expression of SLA/LP and RXFP1 proteins is well recognized (Volkmann et al., 2010; Ezhilarasan, 2021; Hu et al., 2021), the expression of these proteins is not restricted to the liver, and little is known about the tissue expression of the isoforms of DIP2A targeted by autoantibodies identified by PhIP-seq. However, DIP2A was the only protein among the top hits that enriched for three overlapping peptides; these three peptides share a region of 19 identical amino acids (Fig. 3 a). Given that any peptide enrichment may be the result of antibodies directed against host proteins, or alternatively against similar nonhost sequences, such as a virus, we performed a protein-level BLAST search using the enriched motif as the query. Of the viruses that infect humans, the top match to the DIP2A region of overlap was the U27 protein of human herpesvirus 6 (HHV6; Fig. 3 a). U27 is a viral protein required for replication, which enhances processivity of the viral DNA polymerase, key to the HHV6 lifecycle (Poetranto et al., 2020). The region with sequence similarity to DIP2A is found in the N terminus of U27 (Fig. 3 b) and is identical in both HHV6A and HHV6. Interestingly, the SLA/LP protein has also been previously reported to have 41% homology to a peptide derived from the HHV6 U14 protein (Herkel et al., 2002). We note that a BLAST search for identical matches to the 9–amino acid motif in the HHV6 U27 protein identified seven other microbes that are either known to infect or colonize humans, are potentially associated with human hosts, or are of unknown host range (Table S3). Of these results, only HHV6 is a common human pathogen.

To identify whether antibodies from DIP2A-positive AIH patients react with U27, a PhASER library was employed to display a 49–amino acid stretch of HHV6 U27, spanning the region of similarity. A series of 12 mutated peptides were designed featuring a moving window of six consecutive alanine residues, tiled by 3–amino acid steps (Fig. 3 c). Robust enrichment was observed for the wild-type HHV6 peptide sequence among DIP2A-positive patients (Fig. 3 c), not seen in healthy controls. In contrast, when alanines spanned positions 4–30, this enrichment was ablated. Amino acids 4–30 directly encompass the putative similarity region between HHV6 and U27 (residues 8–16), identifying this region as central to antibody binding. The critical region of HHV6 U27 highlighted by the PhASER library has several notable features. First, residues 9–23 are composed of repeating HR[D/E] triplets. Second, BepiPred 2.0 prediction (via https://iedb.org/) of linear B cell epitopes within the U27 protein surfaced residues 4–26 as a top-ranked candidate (yellow bar, Fig. 3 e), a prediction validated by our empirical evidence of antibody binding to a linear epitope in that region.

**Figure S1.**
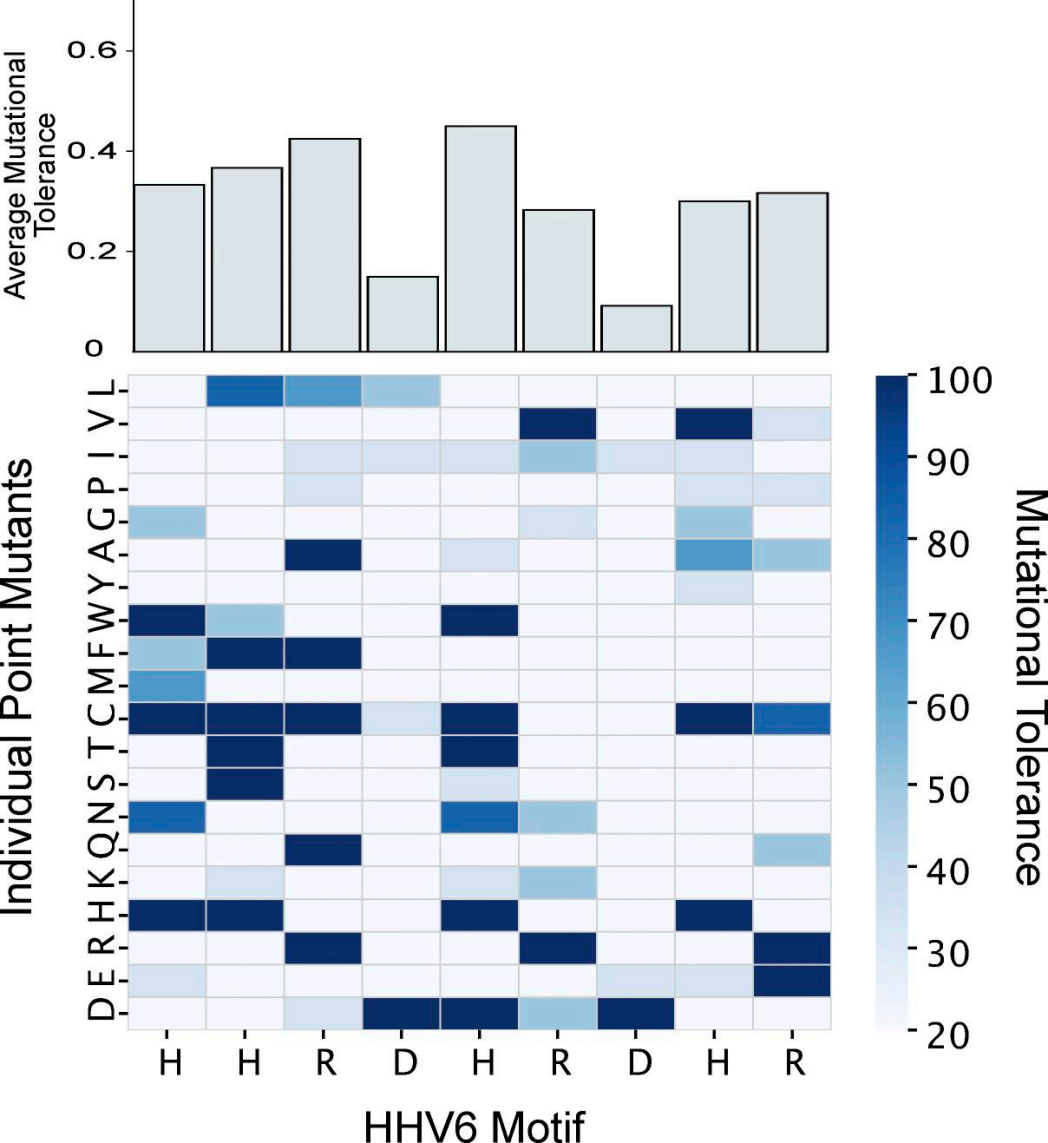
**Deep mutational scanning of the cross-reactive motif between DIP2A and HHV6 U27.** The heatmap denotes the impact of point mutation at each position in the motif HHV6 U27, residues 8–16 (x axis) relative to each point mutant (y axis), which are grouped by properties of their side chains (1, acidic/negative charge; 2, basic/positive charge; 3, polar, uncharged; and 4, hydrophobic). Data are expressed as a percentage of the wild-type U27 reactivity (mutational tolerance, legend at right). Average mutational tolerance at each position of the motif is summarized at the top of the heatmap.

**Figure S2.**
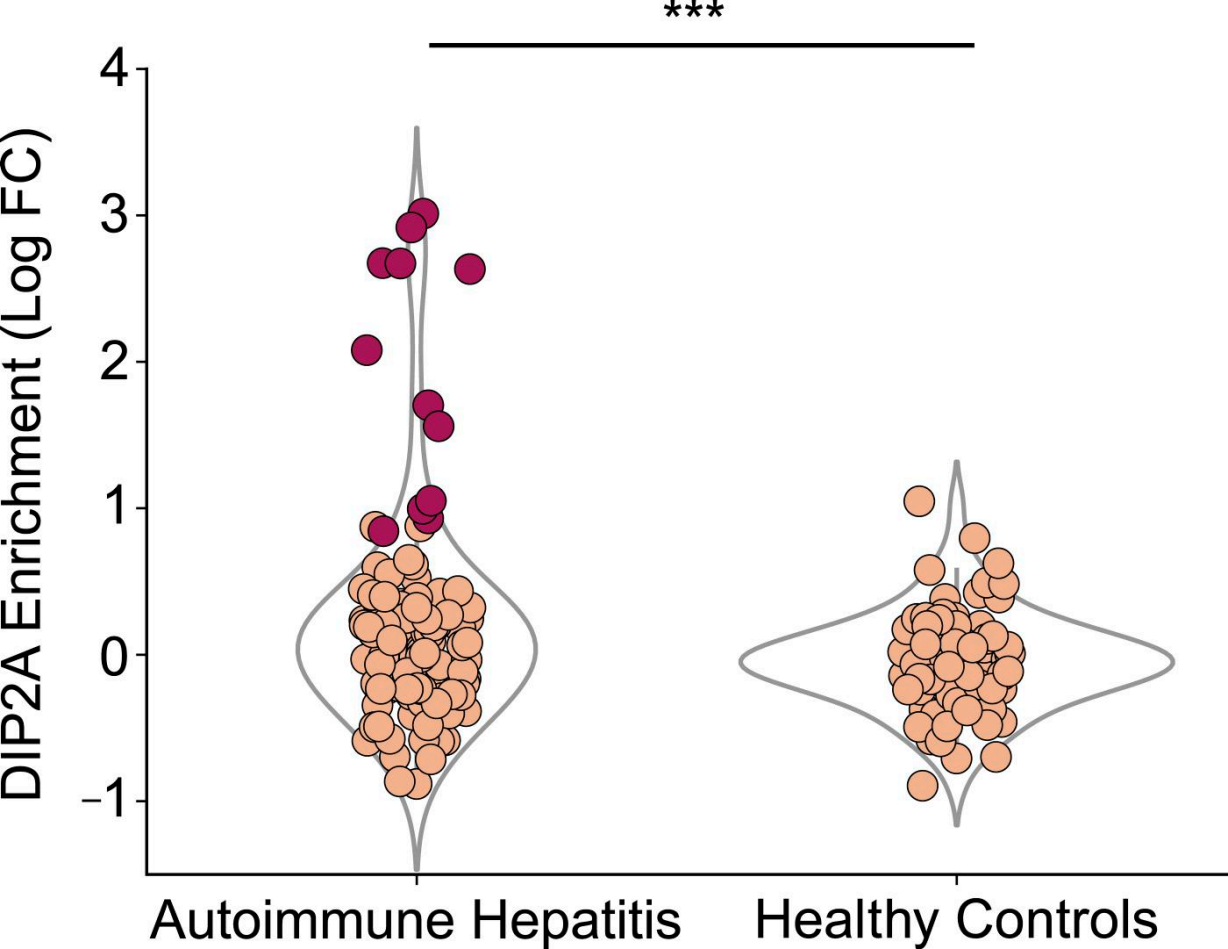
**Enrichment in DIP2A by PhIP-seq.** Enrichment in AIH versus healthy controls (x axis), measured by log fold change (log FC) of DIP2A relative to the mean of DIP2A enrichment in healthy control samples (y axis), where patients with a Z-score of >3 relative to the mean of healthy controls are colored in dark red (***P < 0.01).

To further investigate the contribution of individual amino acid differences in the region of putative cross-reactivity between DIP2A and the HHV6 U27 sequence (residues 8–16), we used PhASER to perform deep mutational scanning to identify precise determinants of antibody binding, encoding all possible single-point mutants at each position of this region (Fig. S1). This analysis highlighted the importance of the acidic residues. Focusing this approach among the point mutants, the two nonidentical positions were mutated from the HHV6 sequence to the DIP2A sequence (Fig. 3 d). As expected, the semi-conservative change from histidine to glutamine had only a minor impact on immunoprecipitation enrichment with patient sera, whereas the nonconservative change from aspartate to threonine nearly abolished enrichment. These results are consistent with the notion that the true target epitope of these antibodies in AIH patients derives from HHV6. Furthermore, no healthy control patients reached our significance threshold for reactivity against DIP2A (z > 3, Fig. S2 a).

To further investigate the hypothesis that antibodies from DIP2A-positive patients target HHV6, we developed a customized competition Luminex assay where U27 or DIP2A peptides conjugated to BSA, or BSA-only reference controls, were covalently linked to Luminex beads. These conjugates were incubated with patient serum directly as previously reported (Zamecnik et al., 2024). Peptide competition experiments were performed by adding serum that was preincubated with the competitive peptide; for all conditions, normalized reactivity was calculated as the ratio of peptide–BSA reactivity to BSA reactivity alone (Fig. 3 e). Preincubation with the putative cross-reactive peptide decreased reactivity (relative to negative control, BSA-only reactivity); the competition results indicate the U27 peptide is a superior blocker of DIP2A reactivity, when compared to the impact of DIP2A preincubation on HHV6 reactivity. This is consistent with the mutagenesis data shown in Fig. 3 d, which suggests that HHV6 is the dominant epitope.

HHV6 establishes latency in the liver, reactivation has been associated with hepatitis (Phan et al., 2018), and primary HHV6 infection has been previously associated with the onset of AIH (Schmitt et al., 1996). To investigate for evidence of HHV6 infection, and evaluate the possibility of reactivation, we performed HHV6 serologic profiling in AIH patients positive or negative for DIP2A antibodies, as well as in healthy controls. These data demonstrate a ∼90% seroprevalence of HHV6 IgG, as anticipated, given >90% of patients in developed nations are exposed to HHV6 by the age of 3 (Agut et al., 2015). However, AIH patients positive for DIP2A had a greater titer of anti-HHV6 IgG. The reactivation of HHV6 has been linked to higher HHV6 IgG titers (Dahl et al., 1999) but not IgM (Tohyama et al., 1998); this result is suggestive of HHV6 reactivation among AIH patients positive for DIP2A.

### Orthogonal validation of RXFP1 and correlation with patient metadata

Relaxin-2 signaling through RXFP1 on the surface of activated hepatic stellate cells has been shown to decrease their fibrogenic potential (Iredale et al., 2017), and relaxin-2 has been used as an antifibrotic agent in clinical trials of patients with alcohol-associated liver disease (Snowdon et al., 2017). Autoantibody-mediated blockade of RXFP1 signaling, which is antifibrotic, has the potential to promote fibrogenesis. Among the nine AIH patients positive for antibodies against RXFP1 by PhIP-seq, eight patients (88%) had evidence of advanced fibrosis (F3 or greater); chronic liver injury and fibrosis are known to induce the expression of RXFP1 on hepatic stellate cells (Snowdon et al., 2017).

**Figure 4.**
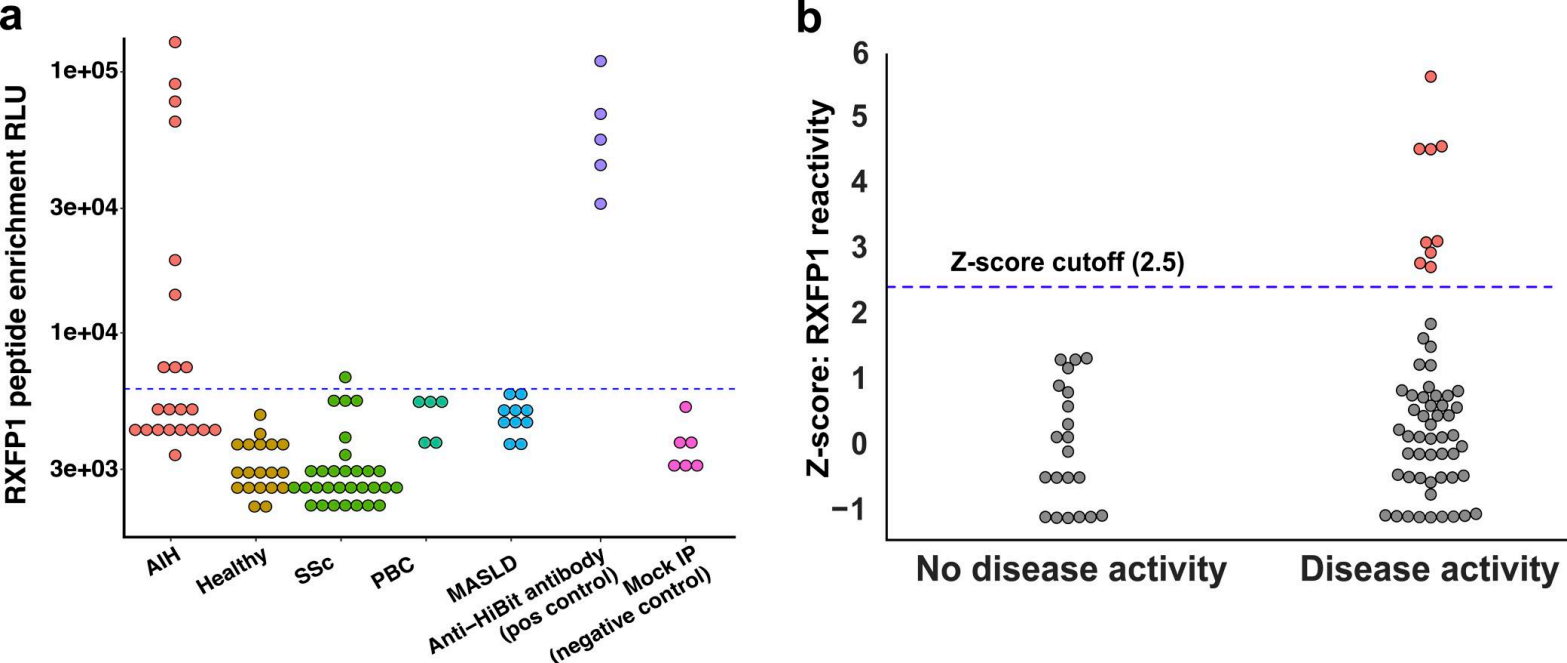
**Orthogonal validation of RXFP1 reactivity, and correlation with AIH disease activity. (a)** SLBA validation of anti-RXFP1 peptide reactivity in various patient groups (x axis), as measured by enrichment of relative light units (RLU, y axis); the cutoff for positivity was set at the mean + 3 SD of all controls (blue dashed line). **(b)** PhIP-seq data plotting Z-score of anti-RXFP1 peptide reactivity among AIH patients (y axis); AIH patients were separated into groups of active versus inactive AIH (x axis); patients considered as positive/reactive against anti-RXFP1 had a Z-score of enrichment >2.5 (denoted by blue dashed line, highlighted in pink).

To validate the RXFP1 finding, we employed a split luciferase binding assay (SLBA), as recently reported (Rackaityte et al., 2023). Briefly, *in vitro* transcription and translation were used to generate the primary RXFP1 peptide identified by PhIP-seq with the addition of a HiBiT tag, which when complexed with the LgBiT protein generates luminescence (Promega system). Immunoprecipitation of tagged peptides was performed with a subset of AIH patients, for which sufficient volumes of serum or plasma were available, in addition to control sera. An antibody targeting the HiBiT protein tag (Promega) was used as a positive control, and negative controls were performed with buffer in the absence of patient serum. A sample was considered positive if the signal in the assay exceeded a cutoff value of the mean + 3 standard deviations from the mean of all control signals (dotted line, Fig. 4 a). This assay demonstrated that the same nine patients positive by PhIP-seq were also positive by SLBA, and none of the liver disease controls or healthy controls were positive (Fig. 4 a). An additional control of another fibrotic autoimmune disease was included, SSc, as relaxin-2 knockout mice develop systemic fibrosis similar to human SSc and relaxin-based therapy has been pursued in clinical trials of SSc as a therapeutic. We found evidence of only a single-positive SSc patient for anti-RXFP1 reactivity among the 30 patients assayed (Fig. 4, green), further suggesting anti-RXFP1 antibodies are associated specifically with AIH as opposed to other autoimmune conditions. Further investigation of the nine RXFP1(+) patients (Z-score >2.5) had some evidence of disease activity (defined by AST or ALT >2× the upper limit of normal or an elevated IgG level), and eight of nine patients (88%) had evidence of advanced fibrosis, F3 or greater.

### Serum from AIH patients with anti-RXFP1 activity inhibits relaxin-2 signaling through RXFP1 in an IgG-dependent manner

**Figure 5.**
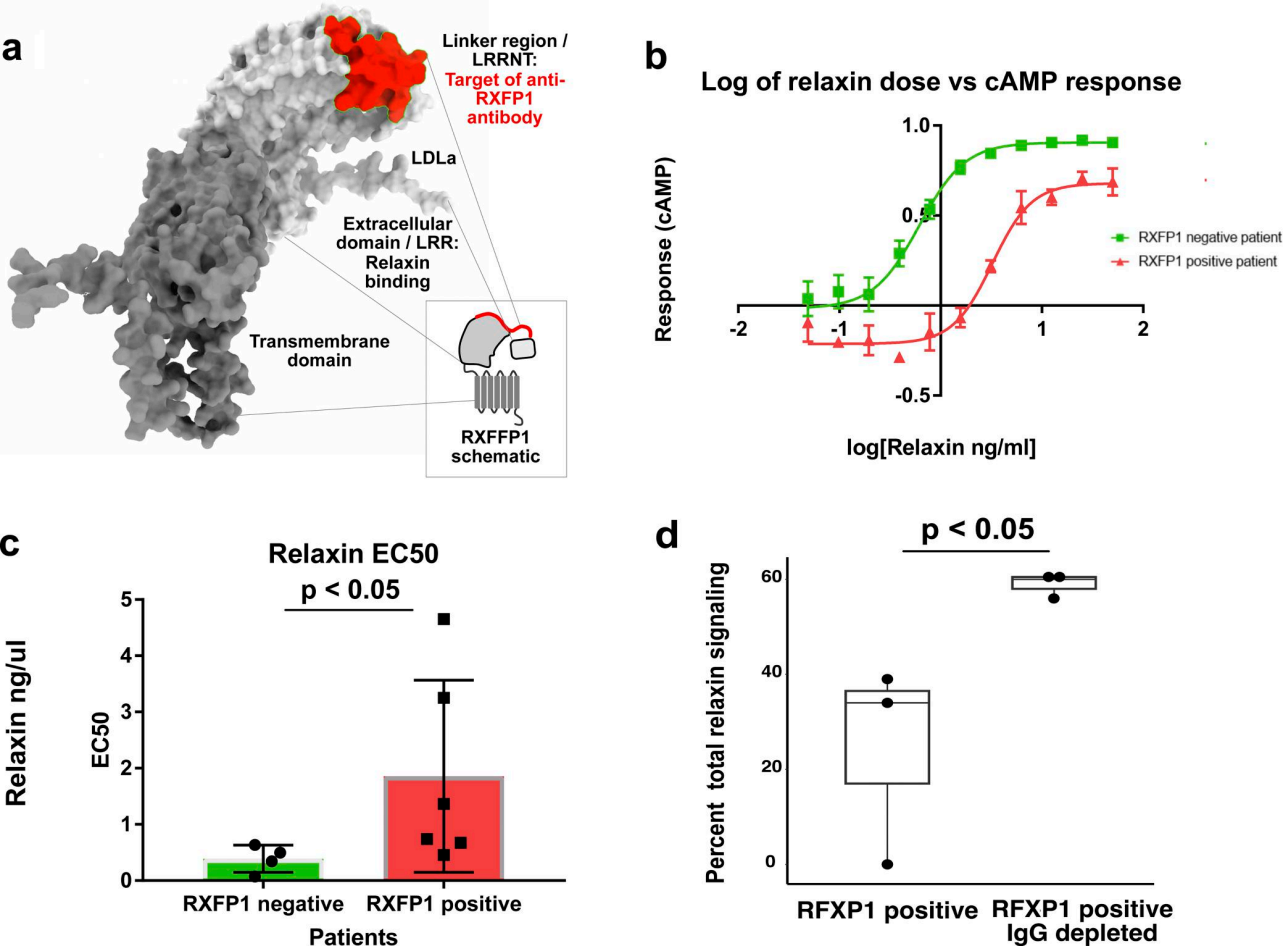
**Serum from AIH patients with anti-RXFP1 activity inhibits relaxin-2 signaling through RXFP1 in an IgG-dependent manner. (a)** Putative structure of RXFP1, as depicted using ChimeraX; the region corresponding to the RXFP1 peptide identified by PhIP-seq is highlighted in red, along with annotation of functional domains (for schematic representation, see panel inset). **(b)** Assay of relaxin-2–induced induction of cAMP by RXFP1, in THP-1 cells preincubated with [1:100] dilution of patient serum negative (green) or positive (red) for RXFP1 antibodies; relaxin concentration, x axis; cAMP response reported as a percentage of untreated control signal, y axis. **(c)** Measurement of relaxin-2 EC50 in ng/μl (y axis) for patient serum negative (green) or positive (red) for RXFP1 antibodies. **(d)** Depletion of IgG using protein A-G beads (x axis, right) or mock-depleted serum (x axis, left) was performed prior to incubating THP-1 cells with patient serum at [1:250]; resultant impact on relaxin-2 signal was expressed as a percentage of untreated signal (y axis).

**Figure S3.**
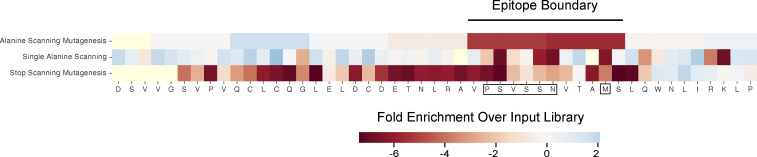
**PhASER epitope mapping of RXFP1.** The RXFP1 peptide is noted at the bottom, with the identified epitope outlined (black box), identified by areas where mutagenesis caused a loss or reactivity (red) as measured by fold enrichment over input library using various approaches denoted on the left axis (single alanine scanning and 6-mer blocks of alanine scanning), as well as stop scanning mutagenesis, used to define the epitope boundary (line above the figure).

The functional implications of RXFP1 positivity were explored further to investigate the possibility that the autoantibody itself is pathogenic in AIH. The proposed structure of RXFP1 is diagrammed in Fig. 5 a using UCSF ChimeraX (Pettersen et al., 2021), with annotation of various domains. The extracellular portion of RXFP1 (light gray, Fig. 5 a) is composed primarily of a large, leucine-rich repeat (LRR) motif, present among a larger family of LRR-containing G protein–coupled receptors (Petrie et al., 2015). This LRR region is the site of relaxin-2 ligand binding. Further highlighted in red is the RXFP1 peptide target of antibodies in patient serum, as identified in PhIP-seq and SLBAs. At the core of this peptide is an LRRNT motif, an N-terminal capping region required to maintain stability of the LRR region. The RXFP1 peptide is at the end of a linker region connecting the LRRNT to a low-density lipoprotein domain (LDLa domain; diagrammed in red, Fig. 5 a inset panel). This linker has been demonstrated to be required for RXFP1 receptor activation (Sethi et al., 2021). Epitope mapping by PhASER allowed us to identify the starting boundary of the epitope by alanine scanning mutagenesis, and the end of the epitope using stop scanning mutagenesis. This approach further narrowed the epitope to a region of seven critical amino acids (Fig. S3). Based on the peptide’s position within RXFP1, we hypothesized that antibodies targeting this epitope would be able to functionally interfere with RXFP1 signaling. We tested this hypothesis using the cAMP-Glo signaling assay (Promega) in which ligand binding to its cognate G protein–coupled receptor (GPCR) leads to the production of cAMP, which can be measured by luminescence as a sensitive read-out of ligand binding to the GPCR. We performed this assay in THP-1 cells, a human monocytic cell line, as cAMP production in response to relaxin-2 binding of RXFP1 has been well characterized in this system (Parsell et al., 1996).

A dose–response curve was first created using THP-1 cells incubated with serum (2 h) from an anti-RXFP1–positive and an anti-RXFP1–negative patient. Treatment with anti-RXFP1–positive serum resulted in a significant shift in the EC50 (fivefold) (Fig. 5 b). Repeating this assay on five additional patients and four additional controls revealed a significant difference over a range of values (Fig. 5 c). AG bead–based depletion of IgG prior to preincubation with anti-RXFP1–positive serum abrogated this effect (Fig. 5 d), consistent with the notion that the inhibitory component in patient serum is indeed IgG immunoglobulins.

In summary, AIH has been rising in incidence and prevalence (Hahn et al., 2023), and despite this trend, the etiopathogenesis of the disease remains incompletely understood. Environmental triggers have been repeatedly implicated in AIH pathogenesis, including infections (Christen and Hintermann, 2014). There are also several experimental models of the AIH that require an infectious trigger to overcome the immune-tolerant environment in the liver (Christen and Hintermann, 2022). However, despite this long-standing association between AIH and pathogens such as HHV6 (Wies et al., 2000), direct molecular evidence linking viral reactivity to AIH is lacking. Furthermore, despite a growing list of biological processes associated with AIH (Adao et al., 2024), advances in disease therapeutics remain elusive (Adao et al., 2024). Novel approaches are required to accelerate our understanding of AIH pathogenesis.

Here, we used PhIP-seq to uncover novel autoantibody targets in AIH patients, by leveraging a large cohort of patients from an international, multicenter collaboration to identify new aspects of AIH pathophysiology. One unique aspect of this cohort is the inclusion of many non-AIH liver disease controls including 178 patients with MASLD. MASLD is a disease that can coexist with AIH, and can be difficult to differentiate from AIH on the basis of serology given the high rates of co-occurring autoantibodies, such as ANA and anti-smooth muscle antibodies, reported to be positive in 20–30% of MASLD patients (Kanzler et al., 1999; Mitra and Ray, 2020). Analysis of the aggregated PhIP-seq data using machine learning, comparing AIH with all controls (including MASLD) by logistic regression, yielded an average AUC of 0.82. These data support the notion that shared autoreactivities exist within subsets of AIH patients. We identified several antibodies highly specific to AIH, which allow differentiation from patients with other liver diseases, including those with MASLD. It is notable that we can detect shared autoreactivities even in patients on immunosuppressive therapy, aligning with previous reports from our group (Bodansky et al., 2024a).

To further leverage the value of PhIP-seq, we focused on the identification of AIH-specific antibodies. SLA/LP, a known autoantigen in AIH, was identified as a highly specific target using this approach, and thus served as an internal control. Using a peptide-based approach, positivity for a single peptide in PhIP-seq was able detect SLA antibody in every patient who clinically tested positive, and with remarkable specificity. Furthermore, the novel targets of autoreactivity we identified in AIH are present at a similar rate to anti-SLA antibodies, but are present in distinct subgroups of patients who are SLA-negative, suggesting the inclusion of novel autoantibodies to DIP2A and RXFP1 in diagnostic assays could increase the detection of additional AIH patients. Notably, none of the targets we identify by PhIP-seq, including SLA, are specific to the liver. Enrichment in autoantibodies targeting DIP2A or RXFP1 was not significantly associated with clinical features of AIH. This novelty may point to uncovered aspect of AIH pathogenesis, which with further study could expand our current understanding of disease-specific phenotypes and elucidate potential pathogenic mechanisms.

RXFP1 has not been previously described in AIH and was enriched by a subset of patient sera, and not in controls. Furthermore, serum from anti-RXFP1–positive patients inhibits relaxin-2 signaling via RXFP1, suggesting the antibodies directed against this protein may play a direct functional role. Specifically, our results suggest that anti-RXFP1 antibodies have potential to diminish the antifibrotic properties of relaxin-2. Additional studies will be required to better understand the role of these antibodies and whether they may enable risk stratification of AIH patients for fibrosis progression.

A third autoreactivity was initially ascribed to the protein DIP2A, which is not known to have a specific role in the liver. Investigation revealed that the autoreactive region of DIP2A shares significant sequence similarity to the U27 protein of HHV6, a common herpesvirus that can achieve latency, and reactivate, in the liver. Through mutagenesis, the critical region was further refined to encompass a 22–amino acid segment near the N terminus of U27. Individual point mutations further support the notion that the observed autoreactivity to DIP2A was due to antibodies directed against HHV6 U27. This segment harbors several triplet repeats, and it also contains predicted linear B cell epitopes. To our knowledge, this is the first evidence that AIH patients positive for highly specific autoantibodies also react with increased reactivity to a highly similar region of viral protein. Furthermore, reactivity to DIP2A could be blocked by preincubating with the HHV6 peptide, consistent with the possibility of antibody cross-reactivity. While the seroprevalence of HHV6 in the adult population can exceed 90% (Saxinger et al., 1988), immunoreactivity to HHV6 was significantly higher among the AIH patients who were DIP2A-positive than AIH patients who were DIP2A-negative or healthy controls. Given that primary HHV6 infection often occurs prior to age 3, well before the onset of AIH-1, we know that HHV6 infection proceeded development of AIH. We hypothesize that HHV6 reactivation could drive AIH onset in a subgroup of DIP2A-positive patients. We cannot exclude the possibility that HHV6 reactivation could instead be a consequence of developing AIH. However, the presence of higher titers of HHV6 IgG among a narrow subset of DIP2A-positive AIH patients argues against this possibility. While beyond the scope of this initial study, further investigation, including studying patients at the time of AIH onset, and T cell epitope profiling using patient peripheral blood mononuclear cells, could yield additional mechanistic insights.

Regarding limitations of this study, the PhIP-seq technique employed here largely captures linear epitopes. Conformational epitopes, posttranslationally modified epitopes, and other nonlinear configurations would likely be undetected in these assays. In addition, analysis by logistic regression suggests that a multitude of features are required to classify AIH samples from healthy subjects, which implies that shared autoreactive targets are common in small subsets of patients. While these data suggest predictive potential, a larger, extensively characterized multicenter validation cohort would be required to further validate these autoantibodies as a clinical diagnostic. Ongoing studies, including additional methods of autoantibody and antiviral antibody repertoire screenings, obtaining samples from AIH patients prior to disease onset, investigation of T cell epitopes, and cloning of antigen-specific B cells from patient PBMCs, would all contribute to a better understanding of AIH and may also help to identify therapeutic targets.

## Materials and methods

### Patient cohorts

This study included both men and women in case and control groups. AIH affects primarily females; this is reflected in the number of female AIH patients studied, as well as in the matched control groups (Table 1). For the current study, biobanked serum or plasma was curated from four established cohorts: POSULD (UCSF, San Francisco, CA, USA), FrAILT (UCSF Parnassus Hospital, San Francisco, CA, USA), the Eppendorf University cohort (Hamburg, Germany), and the UCSF ILD cohort (UCSF Parnassus Hospital, San Francisco, CA, USA). Banked samples included patients with AIH (*n* = 115), MASLD (*n* = 178), PBC (*n* = 26), RA (*n* = 5), and SSc-ILD (*n* = 30), assembled as part of a multicenter, international collaborative effort. All patients with AIH had undergone a liver biopsy, had a simplified IAIHG diagnostic score of probable or definite, and had an appropriate response to therapy. Patients with overlap syndromes or variants were excluded, as were patients with significant autoimmune comorbidities (e.g., RA or systemic lupus erythematosus). For comparison, biospecimens from patients with MASLD were matched by age, sex, race, and with similar prevalence of advanced fibrosis. The diagnosis of PBC and MASLD was made by the primary MD provider, as identified in a chart review. For inclusion in this study, MASLD was defined as (1) having no excessive alcohol use within the last 6 mo, (2) radiological or histological results consistent with steatosis, and (3) without concerning viral (hepatitis B and C virus), metabolic, or autoimmune screen.

Clinical metadata included basic demographic information, laboratory results (including transaminases, IgG, ANA, anti-smooth muscle antibody), fibrosis stage (as assessed by elastography or liver biopsy), and AIH treatment regimen (with or without corticosteroids and duration) at the time of biospecimen collection. Coded specimens were analyzed in a deidentified fashion. Healthy control samples were obtained as de-identified samples from two sources: the first was from the New York Blood Center, as part of retention tubes collected at the time of blood donation from volunteer donors who provided their informed consent for their samples to be used for research; and the second source was plasma from healthy donors obtained from FDA-licensed blood collection facilities, purchased through SeraCare (K2EDTA human plasma).

### PhIP-seq

PhIP-seq was performed as previously reported (Vazquez et al., 2020; Vazquez et al., 2022; Zamecnik et al., 2020), and PhIP-seq protocols described in detail are available at https://www.protocols.io/, with a multichannel-based scaled protocol (https://www.protocols.io/view/scaled-moderate-throughput-multichannel-phip-proto-8epv5zp6dv1b/v1) and a stand-alone guide to library preparation (https://www.protocols.io/view/phage-display-library-prep-method-rm7vz3945gx1/v1).

Briefly, blood from individuals with type 1 AIH and controls was analyzed using PhIP-seq. Phages were cloned to express >700,000 overlapping peptides spanning the human proteome. The following text is adapted from Zamecnik et al. (2024): 96-well, 2-ml deep-well polypropylene plates were incubated with a blocking buffer (3% BSA in TBST) overnight at 4°C to prevent nonspecific binding. Blocking buffer was then replaced with 500 μl of freshly grown phage library and 1 μl of human sera diluted 1:1 in storage buffer (PBS with 0.04% NaN_3_, 40% glycerol, 40 mM HEPES). To facilitate antibody–phage binding, the deep-well plates with library and sample were incubated overnight at 4°C on a rocker platform. 10 μl of each of Pierce Protein A and G Bead (10002D and 10004D; Thermo Fisher Scientific) slurries was aliquoted per reaction and washed three times in TNP-40 (140 mM NaCl, 10 mM Tris-HCl, 0.1% NP-40). After the final wash, beads were resuspended in TNP-40 in half the original slurry volume (20 μl) and added to the phage–patient antibody mixture and incubated on the rocker at 4°C for 1 h. Beads were then washed in RIPA buffer, and then, the immunoprecipitated solution was resuspended in 150 μl of LB-Carb and then added to 0.5 ml of log-phase BL5403 *Escherichia coli* for amplification (OD600 = 0.4–0.6) until lysis was complete (∼2 h) on an 800 rpm shaker. After amplification, sterile 5 M NaCl was added to lysed *E. coli* to a final concentration of 0.5 M NaCl to ensure complete lysis. The lysed solution was spun at 3,220 rcf for 20 min, and the top 500 μl was filtered to remove remaining cell debris. Filtered solution was transferred to a new preblocked deep-well plate where patient sera were added and subjected to another round of immunoprecipitation and amplification, and three total rounds of immunoprecipitation were completed. The final lysate was spun at 3,220 × g for 30 min, with supernatant then filtered and stored at 4°C for subsequent NGS Library Prep. Phage DNA from each sample was barcoded and amplified (Phusion PCR) and then underwent next-generation sequencing on an Illumina NovaSeq Instrument (Illumina).

### SLBA

This assay was performed as recently reported (Rackaityte et al., 2023), and a detailed SLBA protocol is available on protocols.io at https://dx.doi.org/10.17504/protocols.io.4r3l27b9pg1y/v1.

Briefly, the target peptide of relaxin family peptide was identified by PhIP-seq, with the following peptide sequence: 5′-VGSVPVQCLCQGLELDCDETNLRAVPSVSSNVTAMSLQWNLIRKLPPDC-3′. The following text was adapted from Rackaityte et al. (2023): The nucleic acid sequence of this construct was inserted into a split luciferase construct containing a terminal HiBiT tag and synthesized (IDT) as DNA oligomers. Constructs were amplified by PCR using 5′-AAG​CAG​AGC​TCG​TTT​AGT​GAA​CCG​TCA​GA-3′ and 5′-GGC​CGG​CCG​TTT​AAA​CGC​TGA​TCT​T-3′ primer pair. Unpurified PCR product was used as input to rabbit reticulocyte transcription translation system (Promega), and Nano-Glo HiBiT Lytic Detection System (Promega cat no. N3040) was used to measure relative luciferase units (RLU) of translated peptides in a luminometer. Peptides were normalized to 5e6 RLU input, incubated overnight with patient sera, and immunoprecipitated with protein A and protein G Sepharose beads (MilliporeSigma). After thoroughly washing beads with SLBA buffer (0.15 M NaCl, 0.02 M Tris-HCl, pH 7.4, 1% wt/vol sodium azide, 1% wt/vol bovine serum albumin, and 0.15% vol/vol Tween-20), luminescence remaining on beads was measured using Nano-Glo HiBiT Lytic Detection System (Promega cat no. N3040) in a luminometer. Anti-HiBiT antibody (Promega) was used as a positive control for each peptide. A patient was considered positive by SLBA if the RLU exceeded the mean of all controls +3 standard deviations.

### Luminex assay

Luminex assays were performed as previously reported (Zamecnik et al., 2024) with the following modifications. Peptides containing motifs from DIP2A and HHV6 (Table S1) were synthesized by LifeTein and conjugated to BSA via Succinimidyl 4-(N-maleimidomethyl)cyclohexane-1-carboxylate(SMCC) crosslinking at a 1:1 mass ratio. Spectrally distinct Luminex beads were then coupled to the BSA–peptide antigens using xMAP Antibody Coupling Kit (Cat# 40-50016; Luminex) according to the manufacturer’s instructions. A control bead population was prepared by conjugating BSA alone. Conjugations were performed at 5 μg protein per 1 million beads in 0.5 ml reaction volume.

All assays were performed in duplicate, and beads were pooled on the day of use. Thawed serum samples were diluted 1:250 in PBS with 0.05% Tween-20 (PBST) containing 2% nonfat milk and incubated with 2,000–2,500 beads per protein. For preincubation experiments, serum was first incubated with the unconjugated BSA–peptide antigen for 1 h prior to the assay.

During the assay, samples were incubated with beads for 1 h at room temperature with shaking at 250 rpm, washed three times with PBST, and stained with phycoerythrin-conjugated anti-human IgG Fc antibody (Cat# 637310; BioLegend) at 1:2,000 in PBST for 30 min. Beads were washed twice with PBST and analyzed in a 96-well plate format on a Luminex LX 200 cytometer. Net MFI for each peptide was calculated by normalizing to the corresponding intra-assay BSA control and averaging duplicates. Positivity was defined as the mean + 2 SD of negative controls.

### HHV6 IgG ELISA

The HHV6 IgG assay was performed using the Boca Scientific Anti-HHV6 IgG ELISA kit (ODZ-235) according to the manufacturer’s instructions.

### Relaxin-2 signaling/cAMP-Glo assay/IgG depletion

Recombinant human relaxin H2 was purchased from R&D Systems (catalog #6586-RN-025/CF) and resuspended in 1× sterile PBS with 1% BSA at a concentration of 100 μl/ml. In order to block cyclic nucleotide phosphodiesterases during the cAMP-Glo assay, serial dilutions of relaxin were made up in induction buffer composed of 1× PBS with 500 µM 3-isobutyl-1-methylxanthine (Sigma-Aldrich) and 500 µM Ro 20-1724 (Cayman Chemical). Relaxin concentrations ranged from 0.0488 to 50 ng/μl of ligand, and the 12th dilution was “untreated” control, of just induction buffer. Dilutions were made in sterile 96-well plates in order to apply to THP-1 cells to study signaling. THP-1 cells were obtained via ATCC and seeded at a density of 1 × 10^6^ cells/well of a 96-well plate. Prior to the addition of relaxin-2, THP-1 cells were preincubated with patient serum from RXFP1-positive patients or RXFP1-negative patients at a dilution of [1:100] in RPMI with 10% FBS/1% PSG for 2 h in a 37°C incubator. Following this preincubation, relaxin was added at each of the 11 prediluted concentrations to preincubated THP-1 cells, and 96-well plates were returned to the 37°C incubator for 2 h. All reactions were performed in triplicate. Following this incubation, cells were assayed for cAMP production using the cAMP-Glo assay (catalog #V1502; Promega) that was performed per the manufacturer’s instructions, with the following modifications. Lysis of cells was performed using 20 μl of cAMP-Glo lysis buffer for 30 min in standard tissue culture plates and then transferred to opaque white 96-well plates (Nunc) for the remainder of the assay in order to facilitate plate reading of relative light units (RLU) in a luminometer (Promega). The remainder was as per the manufacturer’s instructions. Results were then normalized to the fraction of untreated RLU, and EC50 values were calculated using GraphPad Prism software. For depletion experiments, prior to the cAMP-Glo assay, as described above, sera were preincubated for 2 h at room temperature with Pierce Protein A and G Beads (Pierce) at a ratio of 20 μl of A/20 μl G to 1 μl of serum, with gentle rocking. Samples were ultimately preincubated with THP-1 cells at a dilution of [1:250] prior to the addition of relaxin-2.

### Statistics and bioinformatics analysis

Raw sequencing reads were aligned to the input peptide library using RAPSearch2 (protein alignment), as previously described (Vazquez et al., 2020). Aligned reads were controlled for varying read depths by normalizing RPK. Normalized PhIP-seq read counts were further analyzed using Python. Code to perform phage data analysis can be found in the PhagePy package (https://github.com/h-s-miller/phagepy). Briefly, fold change for each peptide was calculated relative to the mean RPK of healthy controls, and Z-scores were derived from the background distribution. A peptide was considered a “hit” if its enrichment exceeded three standard deviations above the mean of healthy controls, was present in at least 6 AIH patients (∼5% sensitivity), and was observed in no >1% of control patients (99% specificity).

Logistic regression analyses were performed using the Scikit-learn package in Python (50), employing the liblinear solver with L1 regularization. Model performance was evaluated using fivefold cross-validation with an 80/20 train/test split. Molecular graphics and structural analyses were conducted with UCSF ChimeraX, developed by the Resource for Biocomputing, Visualization, and Informatics at UCSF, supported by National Institutes of Health (NIH) grant R01-GM129325 and the Office of Cyber Infrastructure and Computational Biology, National Institute of Allergy and Infectious Diseases.

For ANOVA, statistical analyses were conducted using Python (statsmodels). Univariate analyses assessed associations between each molecular outcome and clinical or laboratory predictors. The Z-score of enrichment was used as the outcome variable for each top hit (DIP2A_58, DIP2A_59, DIP2A_60, RXFP1_4, RXFP1_5, SEPSECS_18, SEPSECS_19) and compared with relevant clinical predictors summarized in Table 2, including therapy type, fibrosis stage, and biochemical measures (AST, ALT, IgG). Outcome variables were treated as continuous, and predictor variables were analyzed according to their natural type (categorical or continuous). To account for multiple comparisons across outcomes and predictors, P values were adjusted using the Benjamini–Hochberg FDR method. Adjusted P values <0.05 were considered statistically significant.

### Online supplemental material

Fig. S1 shows the results of deep scanning mutagenesis of the HHV6 motif, where each amino acid in the HHV6 motif was substituted for every possible alternative amino acid, and antibody binding by PhIP-seq was reassessed. Fig. S2 shows binding among healthy controls and AIH patients to DIP2A. Fig. S3 demonstrates epitope mapping of the RXFP1 epitope. Table S1 demonstrates the top hits identified by PhIP-seq, and for each peptide, the associated peptide sequence, RefSeq and UniProt ID, sensitivity, and specificity are noted. Table S2 summarizes the complete ANOVA results, comparing clinical AIH-related variables with PhIP-seq hits. Table S3 summarizes the BLAST search results using the HHV6 motif as bait and by identifying human-infecting microbes with 100% sequence identity to the input motif.

## Supplementary Material

Table S1 shows the top 50 peptide hits identified by PhIP-seq.

Table S2 shows ANOVA model results to identify relationships between AIH-related metadata and enrichment in PhIP-seq hits.

Table S3 shows BLAST search results, filtered for known or possible human-infecting microbes with 100% homology to the 9-mer from HHV6.

## Data Availability

PhIP-seq data are available for download at Dryad (https://doi.org/10.5061/dryad.kh18932hj).
